# What Is Important to Older People When Accessing Urgent Health Care: Key Considerations and Recommendations From Consumer Consultations

**DOI:** 10.1111/hex.70311

**Published:** 2025-05-28

**Authors:** Miia Rahja, Sharmilla Zaluski, Leanne Greene, Maria Crotty, Craig Whitehead, Kate Laver

**Affiliations:** ^1^ Flinders Health and Medical Research Institute, College of Medicine and Public Health Flinders University Adelaide South Australia Australia; ^2^ Council on the Ageing Adelaide South Australia Australia; ^3^ Division of Rehabilitation, Aged and Palliative Care Southern Adelaide Local Health Network Adelaie South Australia Australia; ^4^ Caring Futures Institute, College of Nursing and Health Sciences Flinders University Adelaide South Australia Australia

**Keywords:** ambulatory care, consumer consultation, emergency avoidance, emergency service, health services for the aged, hospital, urgent care

## Abstract

**Introduction:**

Emergency departments (ED) worldwide are under pressure. Older people are disproportionally represented in ED, and many of these presentations are for nonlife‐threatening ailments that could be attended to elsewhere. Urgent care services have been established to relieve ED pressures and may be a better alternative for older people with urgent but nonlife‐threatening health issues. The purpose of this study was to understand the needs and preferences of older people when accessing urgent care, and what an ideal journey through an urgent care service would look like.

**Methods:**

This qualitative study consisted of three consumer workshops with engagement activities designed and facilitated by an organisation designed to advocate for the rights and interests of older people in Adelaide, South Australia. Primary analysis was completed using a framework analysis approach, which consisted of identifying key themes and meanings in the audio‐recorded data and workshop outputs (including maps, notes and lists). Regular team meetings were held to discuss findings to enhance study rigour.

**Results:**

A total of 39 participants aged 65 and above took part in the workshops. Most participants were female (*n* = 24, 62%), born in Australia (*n* = 26, 67%) and only spoke English at home (*n* = 36, 92%). Most participants (*n* = 33, 85%) had recent experience with ED. Four themes emerged regarding the needs and preferences for urgent care services: (1) accessible and responsive, (2) age appropriate with expert care, (3) listen to me, my story and (4) safe and well‐planned discharge. Participants felt that there needs to be more information available to the public about urgent care services for older people.

**Conclusions:**

This study has identified needs and preferences of older people when accessing urgent care services. Services should consider these preferences when implementing or refining urgent care services to maximise acceptability.

**Patient and Public Involvement:**

Our workshops engaged service users to explore and articulate their needs and preferences for service development in an urgent care setting for individuals aged 65 and older. These workshops involved public participation to evaluate the currently available services and reflect on their ideal future design. Service user experiences and priorities were the primary data sources. This study underscored the significance of lived experience, aiming to listen, learn and collaboratively reflect to understand and propose ideas for enhancing urgent care through a co‐design process.

## 1 Introduction

High utilisation of emergency departments (ED) is a global problem [1]. Yet, it is estimated that approximately 76% of ED presentations are urgent, but nonlife‐threatening [2]. Many people attend ED because they cannot get an appointment to see their general practitioner (GP; sometimes referred to as primary care physician), or because they require timely diagnostic tests, which may not be available in general practice [3]. Thus, the care that is provided in ED has evolved over the last couple of decades [3]. While ED's were initially established with a focus on cardiac and injury attendances, today they also see a range of other presentations including respiratory, digestive, musculoskeletal, infectious and gastrointestinal diseases, as well as mental health‐related services [2, 4].

In Australia, increasing numbers of older people are utilising ED services [5]. This age group tends to seek care in ED for nonlife‐threatening conditions more often than other age groups [6]. Between 2022 and 2023, 23% of ED presentations were patients aged 65 and over (who make up 17% of the population) [2]. The complex medical and social needs of older people suggests that traditional models of ED care may not be optimal for addressing urgent but nonlife‐threatening conditions [7]. Older people have voiced concerns about their specific needs not being met in busy EDs. Many have described receiving limited assistance with essential tasks such as eating, drinking and toileting, as well as experiencing uncomfortable waiting times [7, 8, 9]. There is also a fear of overnight stays in the ED [8, 9]. Over 50% of older individuals (aged 65 and above) accessing ED are subsequently admitted to inpatient wards [2, 10]. This can result in prolonged hospital stays and associated complications [11, 12]. Extended hospitalisations in older individuals can lead to increased risks of delirium, functional decline, incontinence and falls [13] and a higher likelihood of future readmissions [14].

Various programmes have been developed as an alternative to ED presentation for people with urgent health issues [15, 16]. In Australia, the Government has made significant investments into urgent care services to alleviate pressures on ED [17], including specific urgent care services for older people [9, 18]. In 2025, there were 87 Government funded urgent care clinics that offer Australians free walk‐in service for urgent but nonlife‐threatening conditions, 7 days a week, for extended hours, with no appointment needed [19]. Funding for an additional 50 clinics across the country were announced in March 2025, which brings the total investment to approximately A$658 million [19].

Co‐designing health care services with consumers is key to addressing the gap between research, health‐service provision and health policy [20]. Such co‐design can assist in ensuring that services are acceptable and useful [20]. Thus, it is crucial to ensure that the experience of consumers is at the forefront of planning, delivering and reviewing urgent care services [21]. However, research into the needs and preferences of older people regarding alternative models of urgent care is still lacking. As such, the purpose of this study was to ascertain, through consumer workshops, what older consumers want when accessing urgent care services. The secondary aim was to map out and describe what an ideal journey through an urgent care service would look like.

## 2 Methods

This study is a component of a larger service evaluation of a new ED avoidance model that provides nonemergency but urgent care to people aged 65+ in a six‐bed treatment space. Details of the service have been published elsewhere [9]. The project was approved by the Southern Adelaide Local Health Network Human Research Ethics Committee (ID: 2022/HRE00107).

### 2.1 Context

This study was conducted in collaboration with the Council on the Ageing (COTA) South Australia (SA) social enterprise ‘The Plug‐in’. COTA SA is a not‐for‐profit organisation that advocates for the rights and interests of older people (https://cota.org.au/). The Plug‐in is COTA SA's market insights operation that connects older people with researchers [22]. The Plug‐in consists of a community of people that are demographically diverse and provides older people an opportunity to have a voice that can be used to improve services and policies. Members of the research team worked with the COTA SA's The Plug‐in team to design engagement activities. The consumer workshops were conducted by two facilitators from COTA SA's The Plug‐in, who were experienced in working with older people to gather insights and experiences and knowledge translation. The aim was to elicit information from older people about their needs and preferences for urgent care to evaluate current services and facilitate service development. Three 90‐min workshops each consisted of ‘World Café’ style discussions to generate recommendations for urgent care. World Café is a widely used participatory and qualitative data collection method designed to promote citizen participation and organisational change processes [23]. The method aims to maximise inclusion and facilitate open, informal and intimate discussion to tap into and elicit the views and knowledge present in groups of people [23].

### 2.2 Participants

Participants were informed about the research project and invited to attend workshops by The Plug‐in through electronic communications directly to The Plug‐in's database (older people who register their interest to be involved in research), and through COTA SA's electronic newsletter. Participants were asked to self‐nominate if they were interested. The eligibility criteria were aged 65 and over, or a family member or carer, living in the Southern Adelaide (Australia) catchment area. Although residents of aged care homes were eligible to participate, all participants in this study lived in private homes in the community. All participants received an honorarium in appreciation for their time. All participants provided written informed consent before commencing the workshops.

### 2.3 Data Collection

Each workshop consisted of small group activities to discuss different aspects of urgent care (described in detail in Table S1). The room was split into different activity tables with groups of 4–5 participants. Each table had a facilitator to help guide participants to have meaningful discussions and to complete each activity. After the small group discussions, there were opportunities to feedback to the whole group as the facilitator took notes.

The first activity consisted of participants individually sharing what was most important to them when accessing and receiving urgent care services. A small group discussion was then held at each table to further explore the themes that were shared and to understand the key considerations for urgent care services for older people. The second activity consisted of guiding the participants through an urgent medical scenario in which they completed a ‘journey map’ about what they needed, and wanted, from an urgent care service. The final activity consisted of a case study that asked the participants to think about a hypothetical situation where ‘Ginny’ needed urgent care. The case highlighted some of the common challenges faced by older people when they experience an acute medical episode that requires urgent attention. These situations often involve navigating multiple services, making decisions about whether and where to seek care and managing risks such as falls, isolation or delayed access to appropriate support. The participants were asked to discuss the additional detail they wanted to know to feel comfortable in choosing an urgent care service over being taken to ED. Multimodal artefacts (butcher's paper, sticky notes and whiteboard notes), verbal, written and visual aids were used as prompts. Each workshop was audio‐recorded and transcribed verbatim by a third‐party transcription company. Photographs of completed activities with artefacts were also collected.

### 2.4 Data Analysis

The primary analysis was led by the first study author (M.R.) using a framework analysis approach [24]. The purpose was to understand the needs and preferences of older people in an urgent care situation. Framework analysis is rigorous and transparent and is used to help with evidence‐based policy‐making [24]. The first step included familiarisation with the data. The next step consisted of identifying key themes and meanings that emerged from the workshop discussions. Indexing was then used to assign codes (short words or phrases) to the earlier identified themes. After the themes were identified and indexed, they were summarised into findings. We held research team meetings (M.R., L.G., K.L., and M.C.) that enabled consideration of multiple viewpoints for the interpretation of the data and a wider exploration of the responses. Finally, the findings were considered collectively (including recordings, and other collected materials and artefacts from the workshops), to create a bigger picture of what was discussed in the workshops. These findings were then compared to summaries of findings by COTA SA's The Plug‐in team (S.Z.), which were in agreement. This report adhered to the Standards for Reporting Qualitative Research (SRQR) guideline (see Supporting Information for checklist).

## 3 Results

A total of 39 participants attended the three workshops. Demographic characteristics as well as experience with hospital care and comfort using technology are shown in Table 1. Most participants were Australian born and did not speak a language other than English at home. Over three quarters of the participants (*n* = 33, 85%) had previous experience with ED and agreed that they felt comfortable using technology (*n* = 31, 80%).

**Table 1:** Demographic characteristics of workshop participants.

Demographics	*n* (% of *N*)
Gender	
Female	24 (62%)
Male	15 (38%)
Age group	
65–69	10 (26%)
70–74	5 (13%)
75–79	12 (31%)
80–84	9 (23%)
85–89	2 (5%)
90+	1 (3%)
Country of birth	
Australia	26 (67%)
Other	12 (31%)
Did not specify	1 (3%)
Language other than English spoken at home	
Yes	2 (5%)
No	36 (92%)
Did not specify	1 (3%)
Participants' former experience of hospital care	
Previously presented to an emergency department	33 (85%)
Been to hospital for routine appointments or procedures	27 (69%)
Supported someone else to attend emergency or hospital for routine appointments or procedures	20 (51%)
Participants feel comfortable using technology	
Strongly agree	19 (49%)
Agree	12 (31%)
Neither agree nor disagree	5 (13%)
Disagree	1 (3%)
Strongly disagree	1 (3%)
Did not specify	1 (3%)

### 3.1 What Are the Needs and Preferences of Older People for Urgent Care Services?

All workshops drew upon the participants' experiences regarding what has and hasn't worked when accessing and/or receiving urgent care. Four overarching themes (‘accessible and responsive’, ‘age appropriate with expert care’, ‘listen to me, my story’ and ‘safe and well‐planned discharge’) emerged from these discussions (see Table 2 for topics discussed under each theme and additional quotes). The participants wanted urgent care that is accessible, has skilled professionals to work with their demographic, and supports their quality of life. ED was often seen as the only option for accessing urgent care, for example, because a GP was not available. However, many would risk their wellbeing to avoid ramping (the practice of keeping patients in ambulances outside of ED due to a lack of available treatment spaces within the hospital), long wait times and the chaotic ED environment.

I didn't even go. I rang up and cancelled [the ambulance] because I realise that there's so many people that might probably be worse than me, and so I thought whatever happens, happens … You don't always need to go to hospital. You just need, quite often, advice if you think you've got an emergency. Not being a qualified doctor, I mean, my blood pressure was 210. That to me was urgent. I prayed! I went to bed and hoped I'd wake up in the morning.

(Tanya, Workshop 1)

**Table 2:** Themes to highlight the needs and preferences of older people for urgent care services.

Theme	Topics discussed	Additional quotes
Accessible and responsive	− 24/7 access to medical assistance (not ED) − Close proximity to home − Fast response − Triage on arrival − Access to someone who can troubleshoot the condition, give advice and direct next steps. − Welcoming (‘I'm not an imposition’) − Expectations set on timeframes/waiting period − Regular updates and reassurance	− I think you should be assessed immediately by an RN [registered nurse]… the treatment can come later but you simply want to know what's going on with you. − Well, it needs to be speedy just in case if, uh, the medical attention hasn't been determined that it's not urgent, it needs to be speedy. − I've written the immediate attention when arriving at the hospital. − I just got, uhm, close proximity, uhm, because I think they closed too many centres, you know. − And kept updated, really important just to be advised what's going on. − I think, too, like the treatment needs to be either given there or if not, you're ultimately referred to a hospital. But it should – I feel you should be setting this like 2 h maximum and – and where all relevant tests, x‐rays, everything should be able to be undertaken and I say very complex, like MRIs or something at that centre. − So, it is that waiting period for the carer as well, because they actually have their life too even though, you know, I love my mother, uhm, I do have other – yeah, so other commitments, uh, family or work or whatever.
Age appropriate with expert care	− A quiet, calm and low‐stimulation environment − Comfortable furniture, access to toilet facilities and refreshments − Knowledgeable medical professionals with specialised training to work with older people. − Holistic care – consider mental and spiritual wellbeing, not just the immediate physical needs. − Services in one location, such as pathology and imaging.	− Quiet and calm…And not with the furniture you walk in like in the Emergency Department… Obviously, behind the scenes, it's got to be all professional. − You might need a toilet urgently, instead of lying there. That's very uncomfortable for hours. − Too often you'll find somebody that will come in and attend to what they think is happening and not listen at all to what you're saying, not respect the person there. − Sometimes someone's got to listen to the person who's in distress to ascertain what the issue is before you just – you can't just jump in and take over. − Access to a lot of expertise. So, somebody might come in and it might not be in their field, but they need to have access to whoever they can get to in that field. − Staff have to be specialists and trained for that particular, uh, age group. Uhm, like understanding and do the things that a lot of the kept for past behaviours, you know, like how the doctors used to.
Listen to me, my story	− Use of consistent clear language. − Respectful interactions, and involvement of support people (family/friends) where appropriate. − Respectful, non‐ageist language − Respectful of culture − Listen to my experience and what I know about my own body and medical history. − Find out about me and any surrounding factors that may add stress to situation (e.g., living alone, pets and transport).	− …he said, I won't do it right now; and I said, well when are you gonna do that? …You know I think he's stalling because he's probably saying, Oh, is it worth it? − *Discussion in Workshop 2* − And – and as a senior, to be communicated with a, say, intelligent adult, not as a child. Don't worry little one, you'll be okay. − And you're not everybody's darling. − Yes, don't call us dear or love. That's so disrespectful. − Too often you'll find somebody that will come in and attend to what they think is happening and not listen at all to what you're saying, not respect the person there. − But the other thing is, just because you are elderly, it doesn't mean to say that your abilities, your cognitive abilities are less. − One of the points I put down was respectful of differences, and I thought of myself, because – I mean that's not to – everybody is different and there could be cultural differences or, uhm, religious differences or whatever, all of those kinds of differences.
Safe and well‐planned discharge	− Links to other services are important for assisting in recovery, particularly for individuals with limited supports to assist when they return home. − Involve GP wherever possible. − Using technology or virtual care, where suitable, to support efficient delivery of services, care and recovery.	− Knowing that you're gonna get follow‐up. − The name of somebody [in the service or hospital] to contact if you, perhaps, need some care before that appointment type … so the name and phone number of‐of a person or organisation to contact where you're going to get some immediate or‐or quick response again. − I think a phone call is good. Person‐to‐person, yeah. How – how are you going? Are you managing? Have you got new pain or – yeah, a follow‐up would be helpful. − I guess when, you know, you're going to be going home as well, perhaps there needs to be a pharmacist on site, so that you take your medication – if you have any medication, you can take it home with you, rather than you have to go to the chemist the next day. − *Discussion in Workshop 1:* − I think a phone call is pretty much a waste of time (for follow‐up appointments). Unless it was a video call. − I think that's fine but not everybody – − Not everyone's got a computer – − Not everybody has got Zoom or – − Not everybody has the equipment, nobody knows how to use it, which is a bit of a shame.

#### 3.1.1 Accessible and Responsive

Access to a 24/7 service (i.e., not ED), fast and efficient triage, proximity to home, and prompt attendance to needs were the first items mentioned when the participants were asked about what was important to them when accessing urgent care (Table 2).

I think the key for our age group, going back to my point about 24‐hour, is this one time of the day that we know locums are very hard to get – and GPs won't come out [to your home].

(Dom, Workshop 2)

One participant described a scenario when they needed a GP but could not get an appointment. They chose to pray and wait for an appointment, rather than go to ED.

I called GP, I couldn't get an appointment for five days anywhere… All I wanted was Panadeine Forte and that's what I wanted, a script for that. At the end of five days, I was just lying on the floor, sleeping on the floor in my house because I don't believe in going to emergency—I personally wouldn't go to the emergency for that, because I know that there are more urgent cases, but still, you know.

(Lou, Workshop 1)

The participants wanted a ‘one stop shop’ with access to a multidisciplinary team as well as imaging, pathology, pharmacy and other services on site. This was discussed in Workshop 3 as follows:

Ian: MRIs, scans, – all of the tests that –

Holly: All in the one location.

(Workshop 3)

In addition, although the participants understood that immediate attention may not always be possible in urgent care centres, they emphasised the importance of responsiveness and transparency. One way of doing this would be to set expectations on timeframes and to be kept informed and updated on processes (Table 2). This also included considering the carer and their ability to stay with the patient for a long period of time (Table 2).

If they could say, “Oh you're – you're fourth in the queue”, or something.

(Claire, Workshop 2)

#### 3.1.2 Age Appropriate With Expert Care

The participants wanted to feel safe when accessing urgent care and one participant described that it is important that the staff know how to deal with the underlying anxiety older people may experience in urgent care situations.

It's really important that I think we are feeling safe there.

(Hannah, Workshop 2)

It needs to be attended by someone who knows what they're doing and who knows how to deal with the underlying anxiety.

(Vicki, Workshop 2)

Specifically, an age‐appropriate, quiet and calming environment was important. Comfortable furniture, access to toilet facilities and refreshments were included in features that were valued (Table 2).

Now, when you're very old ‐ as he was ‐ to be in amongst all these people that are either vomiting or noisy, [ED] is not suitable for elderly people.

(Shannon, Workshop 1)

Expert care from staff that are ‘prompt, trained, competent’ (Barb, Workshop 3) was described as important. This included health professionals specifically trained to work with older people, including people living with dementia. The care provided should be personalised, tailored and delivered with compassion (Table 2).

So they'd have to be trained to work with older people, because of all the – you know, there's the hearing, there's the sight, there's the mobility, there's dementia, there's a cultural background, so they'd have to be wanting to, you know, understand all those issues and that's why they're working there.

(Sam, Workshop 3)

#### 3.1.3 Listen to Me, My Story

Clear communication and sharing of information were crucial. Too often the participants felt that decisions were made on their behalf rather than being involved in making decisions about their own care.

I put good communication because, uh, you need to know what's happening when. So when you get into a hospital, like often there's like this long – can be hours. And you have no idea what's happening. So, yeah, uh, it – that's frustrating.

(Claire, Workshop 2)

The participants wanted to be included in decision‐making about their own care and be adequately informed about why certain decisions were made (Table 2). Specifically, ensuring that written and verbal information is presented in clear and consistent way that is easily understood was valued. The distinction between easy‐to‐read information and content that may come across as patronising was emphasised, highlighting the significance of the former. Cultural awareness was also highlighted as an important element of good‐quality urgent care (Table 2).

… they treat you [because you're over 65] that you can't think for yourself, you can't speak for yourself.

(Tracy, Workshop 1)

The participants highlighted that they wanted to be spoken to respectfully. Using terms of endearment was discouraged, and the use of a consumer's own name was preferred (Table 2).

…. ask them if they would like their Christian name or their given name to be used, or would rather their family name because there's a big assumption.

(Robyn, Workshop 2)

They also wanted health care professionals to hear their concerns and understand them as a person.

I want to be treated as a person and not just an illness or an accident, because they – the specialist had been amazing. I'm not faulting them but – and they've cured the cancer or got rid of the cancer, but they are still treating the cancer, they're not looking at me as a person.

(Tessa, Workshop 1)

This included understanding the consumer's perspective of their own health and medical history (Table 2). One participant described it as follows:

Listen to me, [what] my story is all about. Don't make assumptions because [you are a] GP or the triage nurse or whatever.

(Trev, Workshop 1)

#### 3.1.4 Safe and Well‐Planned Discharge

Lastly, the participants discussed how ensuring that a person is discharged home safely was an important part of urgent care.

It's taking some responsibility to ensure that the person has a means of getting home.

(Tracy, Workshop 1)

This included ensuring follow‐up appointments with community GPs were made before discharge and that there were contact details for someone in the urgent care service in case the patient needed to make contact before that appointment (Table 2).

The GP report, so an appointment should be made before the person leaves with the local GP.

(Lou, Workshop 1)

Well, when you're going home, you wanna be able to link back with your GP. And they need to know what's been happening.

(Holly, Workshop 3)

The participants felt that a discharge from an urgent care service should involve family and carers (where requested or appropriate) in the planning process and consider individual circumstances and external supports. Particularly for people who live alone, there was extra worry regarding return to home.

…to contact next of kin then perhaps it would be useful to have that kind of information, too, if they wanted the next of kin to be contacted by the person. Because there may be issues at, you know, at home with family involvement that could, you know, impact upon the situation.

(Trev, Workshop 1)

Using technology and virtual care were raised as treatment options too. However, while the participants expressed a relatively high level of comfort using technology (Table 1), the use of technology for follow‐up consultations was only considered practical if a person had experience with using technology‐based health services (Table 2).

Generally, the older generation would probably not do that [use technology for health care appointments]. You know, they've not grown up with it. They're not going to be comfortable with it. I know a lot of people in our age group that can't do that. They're not computer literate and they don't want to be computer literate because it's all too hard.

(Ruby, Workshop 2)

### 3.2 The Ideal Journey Through an Urgent Care Service

**Figure 1:**
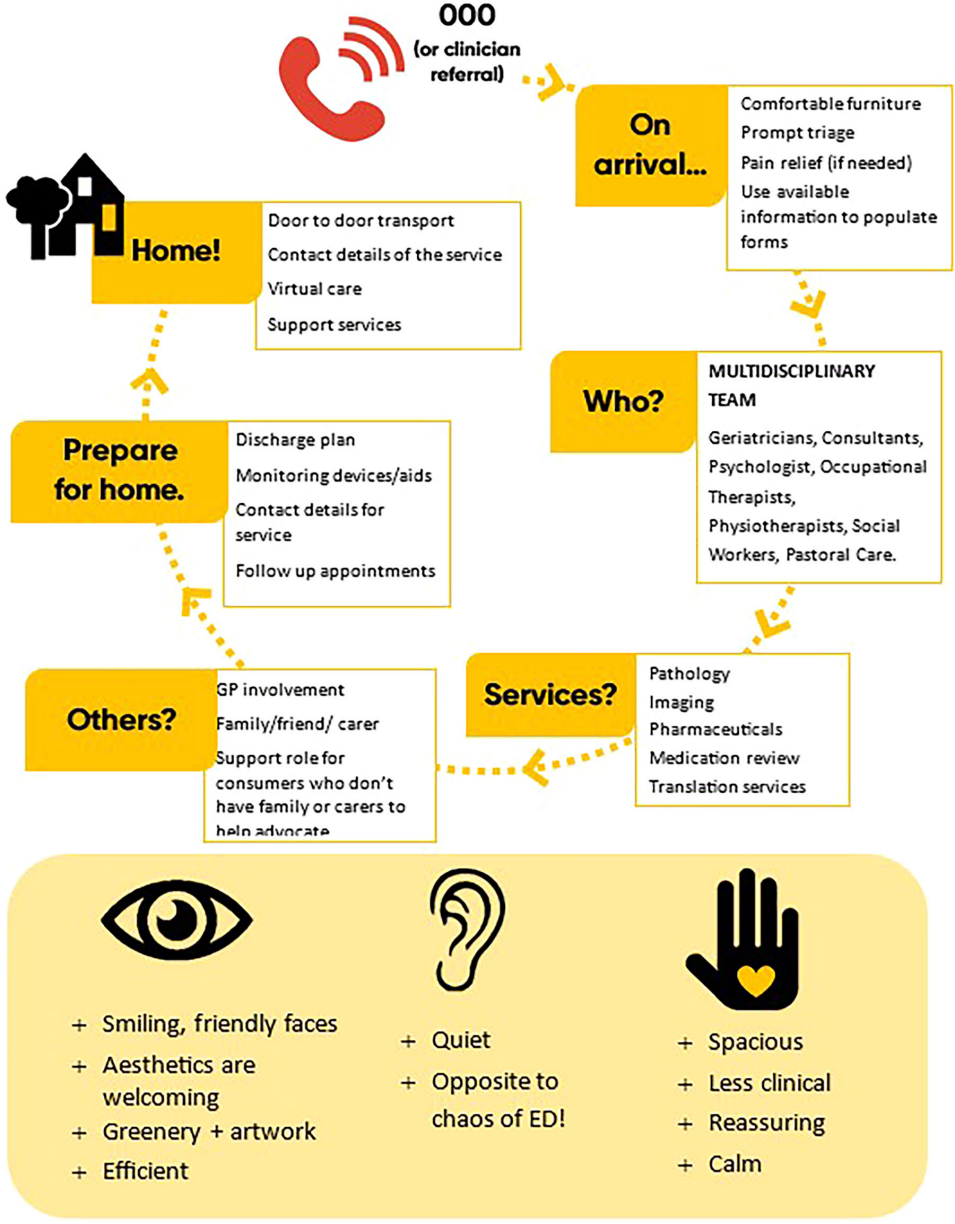
Ideal journey through urgent care.

The ‘ideal journey’ through an urgent health care service as described by our study participants is illustrated in Figure 1 (see Figure S1 for working material). On arrival, prompt and thorough triage was important as well as ensuring comfort and appropriateness of the space for the age demographic. Being seen by a multidisciplinary team with specialised knowledge in care of older people was the next step, followed by access to required imaging, pathology, pharmacy and other services. Inclusion of others, such family and friends were also described as part of the journey, as well as preparation for discharge. The importance of a discharge plan, provision of medications, education on the use of any monitoring devices, referral to support services and ensuring follow‐up appointments were in place were important aspects of this step.

### 3.3 What Information Is Needed for Older Consumers to Feel Comfortable in Attending an Alternative Urgent Care Service Over ED?

The final activity consisted of brainstorming about what information was needed for the participants to feel comfortable in attending an alternative urgent care service over ED for their urgent care needs. Box 1 summarises the key questions posed by participants (information they wanted to know). Overall, the participants felt that there needs to be more information available about urgent care services. Videos and case stories of positive experiences were suggested as useful approaches for creating awareness. Education about the type of care that can be accessed through such services could also improve uptake.

Well, there's got to be trust, you know like – I suppose it would be good to see some case studies of people who have taken that opportunity and what they had as an experience.

(Alex, Workshop 3)

Box 1 **Information needed to support choosing the urgent care service.**

1. Is the service specialised to care for older people (aged 65 and above) only?
2. What are the costs related to accessing the service?
3. Is the service public or private?
4. Where is it located?
5. What is the wait time?
6. What medical professionals and services are onsite?
7. Is the service competent?
8. Can I still go to hospital ED, if I needed to?
9. Can family and friends visit?
10. Is the service 24/7, if not, what happens if I am still unwell when it closes?

Knowing that family and friends could visit was considered important.

But I'd also like to‐I'd also like to know that, uh, your family could visit. I think it's very important to carry the family visits as well. So does the facility have provision for visitors?

(Alex, Workshop 3)

Reassurance about continuity of care (e.g., referral to GP) and safe return home were voiced as factors impacting the decision to feel comfortable in attending an alternative urgent care service.

… that person is about to be, uhm, released from that clinic. The family is called – a family contact is made to have everything explained to that family member. So, you know, what's happened, what the prognosis is, what the further treatment is going to be and can someone come and pick the person up. Or do we have to arrange a transport? Will there be someone home or will there be someone to meet the person at the hospital aged care facility?

(Lou, Workshop 1)

Suggestions for reassurance included: making follow‐up contact within 24 h of discharge to check‐in (including the use of virtual care for ongoing support), offering a way for health care users to contact the service if needed, providing discharge summaries to the health care users and GPs, organising transport back to place of residence and reviewing medications.

## 4 Discussion

This qualitative study has described findings from consumer consultation workshops regarding the needs and preferences of older people for urgent care services. We found that older people want timely and efficient urgent care that is provided by staff with expertise in aged care (e.g., with specialist knowledge about dementia). Expectations should be communicated in clear and respectful language and consumers should be included in the decision‐making process about their own care. Our participants designed an ideal journey through an urgent care service. We have also described the key questions posed by older consumers when considering attendance at an urgent care service.

Timely and accessible services were a top priority for our participants, consistent with findings from previous studies exploring older people's preferences for emergency care [25, 26, 27]. In this study, accessibility was primarily characterised by the service's relational and logistical features, which fostered confidence and comfort in seeking timely support close to home. Levesque et al. [28] offer a broader conceptualisation of health service accessibility, outlining five dimensions: approachability, acceptability, availability and accommodation, affordability and appropriateness, which may further explain why older individuals perceive certain services as more accessible than others. This model can further help explain how older people experience and engage with the service. For example, approachability can be influenced by how well the service is known and understood within the community. If people are unaware of the service or unsure of its purpose, they are less likely to use it. Acceptability refers to how well the service environment, culture, and practices align with the values, beliefs, and expectations of service users. This dimension was particularly important to our participants, who highlighted the significance of interpersonal aspects, such as being listened to and treated with dignity, in shaping their comfort with receiving care. Our participants also expressed a preference for care settings that were smaller, quieter, more personal, and removed from the chaotic environment of the ED. These preferences are consistent with other research, where ED have been described as stressful and unsafe for older people [7, 8, 29]. Availability and accommodation were also important to our participants. They valued services that were close to home, offered flexible hours, responded quickly, and were well coordinated with other health providers, particularly through effective discharge planning. In addition, although the service is government subsidised, affordability emerged as a concern during the case study discussion. Thus, it is important that service users are clearly informed about any associated costs to support access.

Patient‐centred approaches that focuses on the health professional‐patient encounters were also identified by our participants as key features of a valued service. Being involved in decision making about their own care has also been reported elsewhere as well as the need for respectful, clear and transparent communication [26]. Terms of endearment, also known as elderspeak, was discouraged by our participants. While other studies have reported that health professionals in ED specifically tailor their communication approach to older people [30], this may be perceived as patronising and disrespectful [31]. Staff competence in technical skills and person‐centred communication is critical to delivering safe, respectful, and effective care. A review by Brickley et al. [32] identified critical components of person‐centred care, including respect and the importance of finding common ground. In an urgent care service for older people, staff expertise in geriatric care can further support positive outcomes. Core competencies for health and aged care workers have been developed and can help ensure care is appropriate and responsive to the needs of this population group [33, 34]. For example, competencies may include effective communication with people living with cognitive impairment or recognising and responding to geriatric syndromes such as frailty. These are important factors to consider in service provision, as they promote trust, improve communication, and ensure that care aligns with the values, preferences and needs of patients. Integrating these principles into service design and delivery can enhance both the acceptability and effectiveness of care, particularly for older adults who may face complex health and social circumstances. This underscores the necessity for age‐specific training, informed by service user preferences, in the development of future urgent care services or improvement initiatives.

Our participants described that more needs to be done in terms of promoting urgent care services to the public (in the instance that such service is available in their local area). Many people attend ED because they cannot get appointments with their GP's or because they do not know that urgent care services (specially tailored for older people) exist. Other studies have also found that low triage acuity presentations in ED are often due to issues accessing primary care, namely because a patient does not have a GP, they are unable to get an appointment with a GP, or they have a previous negative experience with a GP [25, 35, 36]. A systematic review found that people choose an ED or urgent care service because of convenience (location, opening hours and no‐cost) and availability of specific treatment and investigation options [36]. Many people lack awareness of other options and attend ED as they feel like they have no alternatives [37]. Urgent care services for older people are a reasonably new concept. Thus, education about these alternative services is needed. Public health campaigns and videos describing consumer experiences and information about local urgent care services could produce value.

Although our findings align with those of other studies concerning the needs and preferences of older adults in emergency and urgent care services [25, 26, 27], numerous challenges exist in implementing these insights. Workforce and resource shortages in health care make it challenging to staff 24/7 urgent care services with professionals who possess expertise in both urgent and geriatric medicine. Consequently, many urgent care centres compromise by operating for extended hours (e.g., 7 am–7 pm). Other desirable features, such as comfortable furniture, timely pain relief and a discharge plan, appear easier to implement. Although the current service evaluation focuses on an urgent care model designed to reduce ED attendance, there also seems to be a demand for urgent care services stemming from barriers to accessing timely primary care services. Future research could investigate the potential structure and characteristics of this type of service.

### 4.1 Strengths and Limitations

A strength of this study was that we conducted three separate workshops to reach our presented study findings. This meant that we captured multiple discussions and viewpoints on the same questions from a range of older people. Our findings are therefore more generalisable for this group in the specific caption area for this study. In addition, this triangulation of data from multiple study informants enabled a richer insight into our topic of investigation and enhanced the rigour of our study [38]. We also had two people from different backgrounds (COTA SA and a senior research representative) analyse and interpret the workshop discussions. A larger research team was involved in interpreting the study findings which is a strength of this study. Lastly, the workshops were conducted by The Plug‐in, COTA SA, who already had an established connection with the study participants. These established relationships, together with our implementation of the World Café approach, aimed to foster a conducive environment where participants felt comfortable sharing their experiences and articulating their needs and preferences for urgent care. A limitation of this study is its single‐city focus within Australia, which may restrict generalisability, particularly to regional areas where access to GP and ED care may differ significantly from metropolitan cities. Additionally, the study population was relatively homogenous, with few participants over the age of 85 years, leading to under‐representation of this age group in our findings. This study also did not have representation from Indigenous Australians. The way this population group views and accesses health care is different to those from nonindigenous background [39], which implies that the findings would not be representative of this population group. However, the broader research programme did include a dedicated work package led by Indigenous Australian researchers. This component supported the use of culturally appropriate research methods, such as yearning circles, to explore their perspectives of urgent care services and to ensure that Indigenous Australian voices were included in shaping their development for older adults in South Australia.

## 5 Conclusion

Older people access ED for nonlife‐threatening conditions more than any other age group. Urgent care services that are designed to address the care needs of these older people could alleviate some of the pressures on ED and are currently implemented in Australia. Implementation of such services should consider the needs and preferences of older people. Older people want urgent care services to be safe, accessible and respectful and be tailored to the unique needs of their age demographic. Availability of diagnostic services, inclusion of family and friends, and community GPs in the discharge planning are key characteristics of a good service and provide reassurance. We have described what an ideal journey through an urgent care service for older people may look like and have laid the foundation for what may be the needs and preferences of older people when accessing urgent care services as an alternative to ED. Larger scale studies that investigate if these priorities are identified across Australia, and elsewhere, are warranted. In addition, future evaluations of urgent care services for older people should consider key outcomes such as appropriate service use, health status and quality of life. Assessing these impacts will help ensure that services do not only align with the needs and preferences of older adults but are also clinically effective.

## Author Contributions

**Miia Rahja:** writing – original draft, investigation, project administration, funding acquisition, methodology, formal analysis, writing – review and editing, validation, conceptualisation. **Sharmilla Zaluski:** data curation, resources, writing – review and editing, formal analysis, visualisation, validation, investigation. **Leanne Greene:** data curation, formal analysis, writing – review and editing, project administration, validation, investigation. **Maria Crotty:** funding acquisition, writing – review and editing, supervision, conceptualisation, investigation, validation, methodology. **Craig Whitehead:** conceptualisation, investigation, writing – review and editing, funding acquisition, validation, methodology. **Kate Laver:** conceptualisation, methodology, investigation, supervision, project administration, writing – review and editing, funding acquisition, resources, validation.

## Ethics Statement

This study was approved by the Southern Adelaide Local Health Network Human Research Ethics Committee (ID: 2022/HRE00107).

## Consent

All participants provided informed consent to participate in this study.

## Conflicts of Interest

The authors declare no conflicts of interest.

## Supporting information

Health Expectations supplementary file 03.

## Data Availability

The data that support the findings of this study are available on request from the corresponding author (M.R.). The data are not publicly available due to their containing information that could compromise the privacy of research participants.
