# Supplementary material for: What Is Important to Older People When Accessing Urgent Health Care: Key Considerations and Recommendations From Consumer Consultations
Source: Health Expect. 2025 May 28;28(3):e70311. doi: 10.1111/hex.70311 (PMC12117259; doi:10.1111/hex.70311)
Supplement: Supplementary file 1 — Health Expectations supplementary file 03. [file HEX-28-e70311-s001.docx]

# Table S1. Details of the World café activities

| **#** | **Objective of activity** | **Instructions for activity** | **Materials used** |
| --- | --- | --- | --- |
| 1 | To establish baseline understanding of urgent health situations. | Individual Activity:  1. Please describe what an urgent healthcare injury or illness is to you.  2. Write down 3 words on the card in front of you that help describe what is most important to you when seeking and receiving urgent medical attention.  Group discussion:  1.Is there a recent time you needed urgent medical attention? How did you go about getting the medical attention you needed?  2. Was it hard to access healthcare services without getting emergency assistance?  3. Can you pinpoint what made it difficult for you to be able to get urgent medical attention?  4. What would have improved your experience? | Individual Activity:  Paper notes for urgent healthcare description  Card and text as for recording 3 words/phrase  Group Activity:  Table Facilitator to collate information and record on large post-it notes |
| 2 | Understanding preferences for health assistance and providers  Does technology help people manage their health?  What are the critical health services needed to provide a holistic urgent healthcare service? | In front of you there is an envelope with images to help prompt your thinking of healthcare services. In this activity, we want to explore how you contact, travel to, and make decisions on which services to access when it comes to maintaining your general wellbeing.  Take 15 minutes to discuss together and complete journey map:   - Communication - Transport and Access? - What services/professionals do you need to be able to access to meet urgent health needs? - What happens after leaving service? | Journey Map:  - A3 Sheet or butcher paper to help participants map out different stages in urgent healthcare scenario.  - Suite of images provided to help prompt thinking and use on the journey map  - Text as to help participants record anything additional or draw additional resources |
| 3 | Troubleshooting scenario to find out how people communicate, mobilise, and access health services  Understand time of day and whether this impacts decisions?  What does the ideal service for a 65+ look like that could help bypass ED? | Case study:  Meet Ginny! Ginny is 68 and lives alone in her home. This morning, she was preparing food in the kitchen and reached high to get an appliance out of her overhead cupboard. Something else slid out at the same time that she didn’t expect which caused her to lose her balance, fall backwards, hurting her ankle and bumping her head on a bench behind her.  Ginny is able to get herself up, but feels shaken, a bit disoriented, and the pain in her ankle is excruciating – she knows she needs to get checked out but can’t easily get herself to health services with her injury.  What we know:   - It’s mid-morning, so generally there are community healthcare centres open at this time of day - She can’t drive herself and can’t walk to the bus stop - She is in pain, needs her ankle and head checked for possible concussion, and possibly some imaging to eliminate a break   Who does Ginny call/where does she go for help?  How does Ginny organise transport?  Who does Ginny need to see?  Do you think Ginny access everything she needs outside of a hospital setting?  What happens after Ginny has received urgent care?  Now think about this happening at a later time of the day, or during the night. Does this change the situation for Ginny?  What would the ideal urgent access and healthcare service look like for Ginny that bypasses presentation to ED for treatment/stay? | PowerPoint to present persona information  Table Facilitator to guide discussion as per activity, collate information and record on large post-it notes |

# Figure S1. The ideal journey


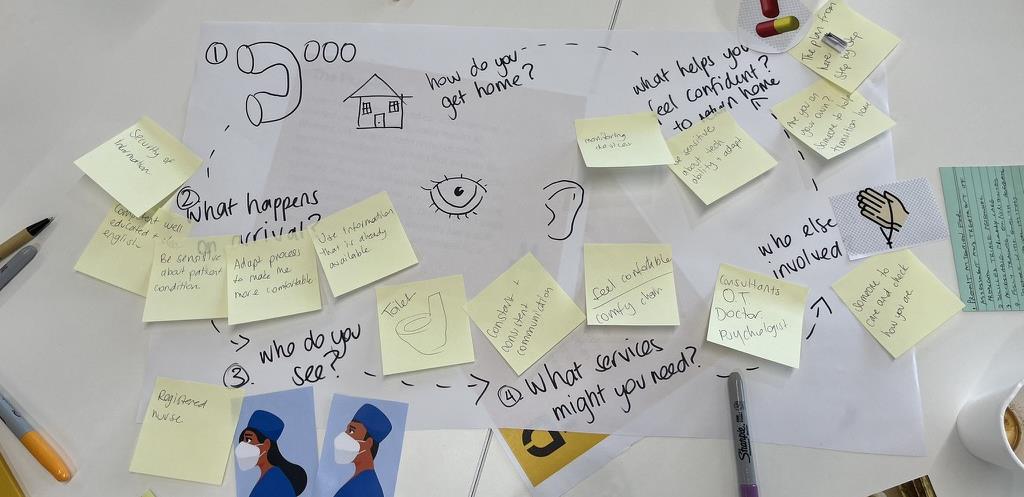


# The Standards for Reporting Qualitative Research (SRQR) - Checklist

| No. | Topic | Item | Tick |
| --- | --- | --- | --- |
| **Title and abstract** | | | |
| **S1** | Title | Concise description of the nature and topic of the study. Identifying the study as qualitative or indicating the approach or data collection methods is recommended. | 🗹 |
| **S2** | Abstract | Summary of key elements of the study using the abstract format of the intended publication. Typically includes background, purpose, methods, results and conclusions. | 🗹 |
| **Introduction** | | | |
| **S3** | Problem formulation | Description and significance of the problem/phenomenon studied; review of relevant theory and empirical work; problem statement. | 🗹 |
| **S4** | Purpose or research question | Purpose of the study and specific objectives or questions. | 🗹 |
| **Methods** | | | |
| **S5** | Qualitative approach and research paradigm | Qualitative approach and guiding theory if appropriate identifying the research paradigm is also recommended; rationale. | 🗹 |
| **S6** | Researcher characteristics and reflexivity | Researchers’ characteristics that may influence the research; potential or actual interaction between researchers’ characteristics and the research questions, approach, methods, results and/or transferability. | 🗹 |
| **S7** | Context | Setting/site and salient contextual factors; rationale. | 🗹 |
| **S8** | Sampling strategy | How and why research participants, documents or events were selected, criteria for deciding when no further sampling was necessary; rationale. |  |
| **S9** | Ethical issues pertaining to human subjects | Documentation or approval by an appropriate ethics review board and participant consent, or explanation for lack thereof; other confidentiality and data security issues. | 🗹 |
| **S10** | Data collection methods | Types of data collected; details of data collection procedures; rationale. | 🗹 |
| **S11** | Data collection instruments and technologies | Description of instruments and devices used for data collection; if/how the instrument(s) changed over the course of the study. | 🗹 |
| **S12** | Units of study | Number and relevant characteristics of participants, documents or events included in the study; level of participation (could be reported in results). | 🗹 |
| **S13** | Data processing | Methods for processing data prior to and during analysis. | 🗹 |
| **S14** | Data analysis | Process by which inferences, themes, etc. were identified and developed; usually references a specific paradigm or approach; rationale. | 🗹 |
| **S15** | Techniques to enhance trustworthiness | Techniques to enhance trustworthiness and credibility of data analysis; rationale. | 🗹 |
| **Results/findings** | | | |
| **S16** | Synthesis and interpretation | Main findings; might include development of a theory or model or integration with prior research or theory. | 🗹 |
| **S17** | Links to empirical data | Evidence to substantiate analytic findings. | 🗹 |
| **Discussion** | | | |
| **S18** | Integration with prior work, implications, transferability, and contribution(s) to the field | Short summary of main findings; explanation of how findings and conclusions connect to, support, elaborate on, or challenge conclusions of earlier scholarship; discussion of scope of application/generalizability; identification of unique contribution(s) to scholarship in a discipline or field. | 🗹 |
| **S19** | Limitations | Trustworthiness and limitations of findings. | 🗹 |
| **Other** | | | |
| **S20** | Conflicts of interest | Potential sources of influence or perceived influence on study conduct and conclusions, how these were managed. | 🗹 |
| **S21** | Funding | Sources of funding and other support; role of funders in data collection, interpretation and reporting. | 🗹 |

O’Brien, B.C., Harris, I.B., Beckman, T.J., Reed, D.A., & Cook, D.A. (2014). Standards for Reporting Qualitative Research: A synthesis of recommendations. *Academic Medicine*, *89*(9), 1245-1251. doi: 10.1097/ACM.0000000000000388
